# C-reactive protein provides superior prognostic accuracy than the IMDC risk model in renal cell carcinoma treated with Atezolizumab/Bevacizumab

**DOI:** 10.3389/fonc.2022.918993

**Published:** 2022-08-01

**Authors:** Ahmad Y. Abuhelwa, Joaquim Bellmunt, Ganessan Kichenadasse, Ross A. McKinnon, Andrew Rowland, Michael J. Sorich, Ashley M. Hopkins

**Affiliations:** ^1^ College of Medicine and Public Health, Flinders University, Adelaide, SA, Australia; ^2^ Beth Israel Deaconess Medical Center, Harvard Medical School, Boston, MA, United States; ^3^ Department of Medical Oncology, Flinders Centre for Innovation in Cancer, Flinders Medical Centre, Adelaide, SA, Australia

**Keywords:** C-reactive protein, renal cell carcinoma, survival prognosis, immunotherapy, IMDC model

## Abstract

**Background:**

Immune checkpoint inhibitors (ICIs) is the main treatment option for patients with metastatic renal cell carcinoma (mRCC); however, significant heterogeneity in response is commonly observed. This study aimed to evaluate the ability of C-reactive protein (CRP) to predict overall survival (OS) and progression-free survival (PFS) in patients with mRCC treated with immunotherapy.

**Patients and Methods:**

Data from patients with mRCC treated with atezolizumab plus bevacizumab in the IMmotion150 and IMmotion151 trials were pooled. Cox proportional regression was used to model prognostic associations. The relative importance of CRP against International Metastatic RCC Database Consortium (IMDC) factors was confirmed using machine learning.

**Results:**

CRPs were available from 527 patients (mean[range] CRP, 6.3[0.21–340]mg/L). Elevated CRP was significantly associated with worse OS (HR[95%CI], 1.71[1.54–1.90], p<0.001) and PFS (1.27[1.18–1.35], p<0.001). CRP was the most prognostic factor for survival within the available clinicopathological data. The prognostic performance of CRP was superior to IMDC model for OS (CRP c=0.76, IMDC c=0.67, p<0.001) and PFS (CRP OS c=0.62, IMDC c=0.59, p=0.03). Predicted 2-year OS probabilities for patients with CRP values of 0.5, 5, 40, and 150 mg/L were 96%, 73%, 42%, and 23%, respectively.

**Conclusions:**

CRP is a powerful prognostic marker for survival, and its prognostic value was superior to the IMDC risk model. This study highlights that CRP could be implemented as stratification factor for mRCC immunotherapy trials and potentially as an easy-to-use prognostic tool in the clinic.

## Introduction

Renal cell carcinoma (RCC) is the most common kidney malignancy, with approximately 20% of patients presenting with metastatic disease at diagnosis (1). Immune checkpoint inhibitors (ICIs) are an established treatment option for metastatic RCC (mRCC). ICIs target the programmed death 1 (PD-1) or the PD-ligand 1 (PD-L1) pathway to remove cytotoxic T-cell inhibitions weakening antitumor immune responses (2). However, at present, there are no markers that accurately predict/prognosticate clinical outcomes of ICI treatment, and significant heterogeneity in response to ICIs remains between patients (2, 3). Therefore, prognostication of clinical outcomes for patients treated with immunotherapy remains of significant clinical interest.

Tumor-associated systemic inflammatory response, consisting of overexpression of proinflammatory cytokines, plays a critical role in cancer cell proliferation, angiogenesis, and metastasis (4, 5). C-reactive protein (CRP) is a clinicopathological marker of systemic inflammation and immune activation and can be readily measured in peripheral blood samples. Elevated CRP has been demonstrated to be a poor prognostic marker in many cancers including mRCC (6–9). While the underlying mechanisms are not yet fully understood, studies suggest that high CRP is correlated with an immunosuppressive tumor microenvironment *via* the infiltration of immune suppressor cells (including regulatory T cells and tumor-associated microphages) (10, 11). Coincidingly, it is hypothesized that elevated CRP may be associated with a downregulation of the antitumor immune responses of ICIs due to its correlation with immunophenotypes of ICI resistance, tumor growth, and poor prognosis (10, 11). These hypotheses are supported by recent studies highlighting CRP as the most prognostic clinicopathological marker for survival in non-small cell lung cancer (n=751) (12) and urothelial cancer (n=896) (13) cohorts treated with the ICI atezolizumab. As data on the prognostic significance of CRP in patients with mRCC initiating ICI are limited, we sought to investigate the prognostic significance of CRP in patients with mRCC treated with immunotherapy. The prognostic significance of CRP was studied in a combined two clinical trial cohorts of patients receiving atezolizumab plus bevacizumab.

## Materials and methods

### Study population

Individual participant data from randomized phase 2 IMmotion150 (ClinicalTrials.gov identifier: NCT01984242) and phase 3 IMmotion151 (ClinicalTrials.gov identifier: NCT02420821) clinical trials were utilized for this *post-hoc* analysis. IMmotion150 involved patients with untreated mRCC randomized 1:1:1 to receive atezolizumab (1,200 mg IV every 3 weeks) with or without bevacizumab (15 mg/kg IV every 3 weeks) versus sunitinib (50 mg orally once daily for 28 days of each 6 weeks cycle) (14). IMmotion151 randomized patients with untreated mRCC to receive atezolizumab (1,200 mg IV every 3 weeks) plus bevacizumab (15 mg/kg IV every 3 weeks) versus sunitinib (50 mg orally once daily for 28 days of each 6 weeks cycle) (15).

Data were accessed according to the Hoffmann–La Roche policy and has been made available through Vivli, Inc. (www.vivli.org). Secondary analysis of de-identified data was confirmed exempt from review by the Southern Adelaide Local Health Network, Office for Research and Ethics, as it was classified as negligible-risk research.

### Predictors and outcomes

The primary evaluated outcome was overall survival (OS). Progression-free survival (PFS) was a secondary outcome. OS was defined as the time from randomization to the last follow-up or death from any cause. PFS was assessed by the investigator per the Response Evaluation Criteria in Solid Tumours (RECIST) version 1.1 (14, 15).

The primary assessed covariate was baseline CRP. The International Metastatic RCC Database Consortium (IMDC) risk tool and pre-treatment levels of hemoglobin, neutrophils, platelets, corrected calcium, and Eastern Cooperative Oncology Group Performance Status (ECOG PS) were available.

### Statistical analysis

The atezolizumab plus bevacizumab arms within IMmotion150 and IMmotion151 were used in this *post-hoc* analysis. Cox proportional hazard regression was used to assess the prognostic associations with OS and PFS. Results were reported as hazard ratios (HRs) with 95% confidence intervals (95% CIs). Continuous variables were explored for potential non-linear associations using restricted cubic splines, and skewed data were log-transformed. Prognostic performance was evaluated using the c-statistic (c) by Harrell (16).

The IMDC risk tool was developed and validated for patients receiving antivascular endothelial growth factor therapy (anti-VEGF) (17). The prognostic performance of CRP was compared to IMDC risk tool and its individual factors (i.e., hemoglobin, neutrophils, platelets, corrected calcium, and ECOG PS). The relative importance of CRP against IMDC factors was confirmed using a machine learning random forest approach (18). The relative importance of variables in the random forest model was determined using a permutation variable importance measures (19), where, on a scale of 0–100, the prognostic strength of a variable is represented. All analyses were stratified, and statistical significance was set to p < 0.05.

Survival probability curves were predicted using flexible parametric survival analysis (20). Exploratory analysis on the sunitinib arms was conducted. A new interactive web-based application incorporating the CRP-prognostic model was developed using the Shiny R package (21). A sensitivity analysis for assessing the prediction performance of CRP using optimal cut points was conducted. Optimal cut points were selected based on maintaining the best discrimination performance compared to using the continuous predictor. All analyses were conducted using the R statistical environment (version 3.6.2).

## Results

### Study population

A total of 552 patients were randomized to atezolizumab plus bevacizumab within IMmotion150 and IMmotion151 trials, of which 527 (95%) had available pre-treatment CRP (median CRP [range], 6.3 [0.21–340] mg/L). Pre-treatment patient characteristics are presented in **Supplementary Table S1**. Pre-treatment patient characteristics according to CRP optimal cut groups is presented in **Supplementary Table S2**. Median (95% CI) follow-up was 19 (18–19) months within the atezolizumab plus bevacizumab cohorts.

### Prognostic significance of CRP with survival

**Figure 1:**
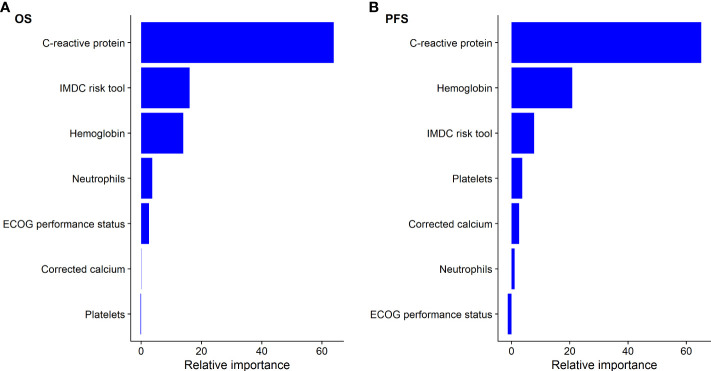
Relative importance of C-creative protein to IMDC factors for predicting **(A)** overall survival (OS) and **(B)** progression-free survival (PFS) using random forest for patients treated with atezolizumab plus bevacizumab.

The continuous associations of CRP, neutrophils, hemoglobin, platelets, and corrected calcium with OS and PFS within the cohort of patients initiated on atezolizumab plus bevacizumab was best described *via* a log-linear relationship. Elevated CRP was significantly associated with worse OS (log-CRP HR [95% CI], 1.71 [1.54–1.90], p < 0.001) and PFS (1.27 [1.18–1.35], p < 0.001) (**Table 1**). According to the c-statistic, elevated CRP was identified as more prognostic than neutrophils, hemoglobin, platelets, corrected calcium levels, and ECOG PS for OS and PFS (**Table 1**). This finding was validated using a random forest approach, which similarly ranked CRP as the most prognostic variable for OS and PFS (**Figure 1**).

**Table 1:** Prediction performance and effect size of the association of C-reactive protein and IMDC risk tool with overall survival and progression-free survival for patients treated with atezolizumab plus bevacizumab.

	Overall survival					Progression free survival				
	n	HR	95% CI	p-value	c	n	HR	95% CI	p-value	c
Log C-reactive protein (mg/L)	527	1.71	1.54–1.90	<0.001	0.76	527	1.27	1.18–1.35	<0.001	0.62
IMDC risk group	552			<0.001	0.67	552			<0.001	0.59
Favorable		1.00					1.00			
Intermediate		2.90	1.73–4.86				1.44	1.11–1.88		
Poor		8.35	4.75–14.7				2.68	1.91–3.77		
Log hemoglobin (g/L)	547	0.05	0.02–0.14	<0.001	0.67	547	0.26	0.12–0.55	<0.001	0.6
Log neutrophils (10^9/L)	543	3.38	2.22–5.14	<0.001	0.64	543	1.87	1.41–2.49	<0.001	0.57
ECOG PS	550	1.89	1.46–2.44	<0.001	0.63	550	1.16	0.98–1.38	0.090	0.53
Log platelets (10^9/L)	547	3.11	2.02–4.80	<0.001	0.62	547	2.11	1.56–2.84	<0.001	0.59
Log calcium, corrected (mmol/L)	441	0.94	0.40–2.18	0.881	0.4	441	1.12	0.59–2.12	0.735	0.56

CI, confidence interval; HR, hazard ratio; OS, overall survival; PFS, progression free survival; IMDC, International Metastatic renal cell carcinoma Database Consortium; n, number of patients; ECOG PS, Eastern Cooperative Oncology Group Performance Status.

Furthermore, the prognostic performance (mean c, 95% CI) for CRP was superior to IMDC risk tool for OS (CRP c=0.76 [0.73–0.80], IMDC tool c = 0.67 [0.63–0.71], p < 001) and PFS (CRP OS, 0.62 [0.59–0.67], IMDC tool c = 0.59 [0.56–0.62], p = 0.03). Demonstrating the higher discrimination of CRP to IMDC risk tool, the 2-year OS probability for the range of reported CRP data (min CRP, 0.2 and max, 340 mg/ml) ranged from 97% to 15%. Comparatively, the 2-year OS probability for the “favorable” versus “poor” IMDC risk groups ranged from 85%% to 33% (**Supplementary Figure S1**). Similar findings were identified within the sunitinib-treated cohort (**Supplementary Table S3**; **Supplementary Figures 2, 3**).

**Figure 2:**
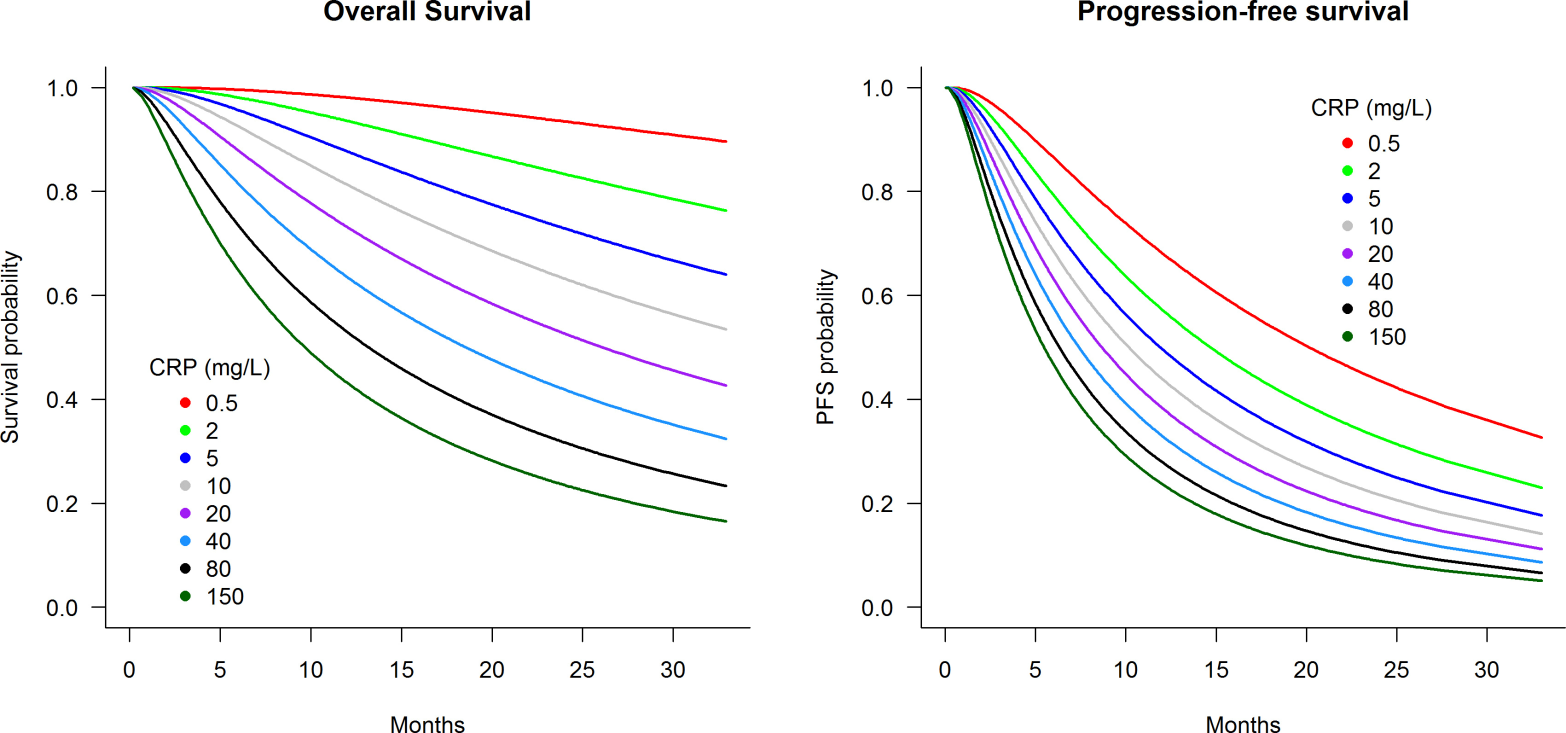
Predicted survival curves according to pre-treatment C-reactive protein level for patients treated with atezolizumab plus bevacizumab.

**Figure 2** presents predicted survival curves of OS and PFS according to pre-treatment CRP levels within the analysis cohort of patients initiated on atezolizumab plus bevacizumab treatment. **Figure 2** demonstrates that pre-treatment CRP levels of 0.5, 5, 40, and 150 mg/L were associated with 2-year OS probabilities of 96%, 73%, 42%, and 23%, respectively, and 2-year PFS probabilities of 44%, 26%, 14%, and 9%. Further predictions of OS and PFS prognosis according to pre-treatment CRP levels can be estimated using the interactive web-based application at https://pmg-flinders.shinyapps.io/crpprognostic/.

As a sensitivity analysis, CRP was optimally cut into three groups (<5, ≥5 <30, and ≥30 mg/L). The HR and prognostic performance of CRP groups are presented in **Supplementary Table S3** and Kaplan–Meir plots in **Supplementary Figure S4**. The OS prognostic performance of CRP groups (c=0.75) was substantially higher than the IMDC risk tool (c=0.67). Furthermore, CRP was associated with significantly reduced objective response rate both when used as a linear predictor (OR [95%CI]: 0.86[0.77–0.96]) and when using CRP optimal cut groups (**Supplementary Table S4**).

## Discussion

Using pooled data from two clinical trials, this study demonstrated that CRP is a strong prognostic marker for survival in patients with mRCC initiating atezolizumab ICI treatment. Furthermore, the performance of CRP as a single factor was demonstrated to be superior to the IMDC risk tool comprised of six factors.

Cancer-related inflammation plays an important role in the progression of tumors and survival of patients with cancer (22, 23). The tumor microenvironment can trigger the release of key proinflammatory mediators including interleukin-6 (IL-6), IL-1, and IL-1β, which stimulate hepatocyte CRP production, leading to a marked increase in plasma CRP (24–26). Furthermore, various studies have demonstrated the ability of RCC cells to locally express CRP, which may also contribute to plasma CRP elevation (27, 28). Elevated CRP has therefore been used as a marker of systemic inflammation with substantial evidence, suggesting that elevated CRP is predictive of poor prognosis in patients with multiple malignancies including mRCC (8, 9, 24). Although the underlying mechanism of CRP correlation with poor prognosis is not fully elucidated, studies suggest that elevated CRP is associated with infiltration of immune suppressor cells including regulatory T cells and tumor-associated macrophages (10, 11). Previous subgroup meta-analysis studies of patients with RCC have identified CRP as a prognostic marker for OS and PFS (6–8). However, the RCC studies included in the meta-analysis were often limited by a small sample size and were based on data from patients who have undergone nephrectomy or were treated with targeted therapies. To our knowledge, the presented study is the first study to compare CRP to the IMDC risk tool and the first to demonstrate CRP as the most prognostic marker for survival in patients with mRCC initiating ICI treatment. This finding is similar to that of prior research for non-small-cell lung cancer and urothelial cancer where CRP was demonstrated as the most prognostic variable in patients initiating ICI treatment (13, 29).

Although including CRP as an additional factor to the IMDC risk tool improved the prognostic performance (c=0.77) compared to the IMDC risk tool (c=0.67), the prognostic performance of CRP as a single factor (c=0.76) was comparable to the combined CRP-IMDC risk model. Therefore, the focus of this paper was on using CRP as a single factor rather than updating the existing IMDC risk model to a seven-factor model.

Currently, there is no recommendation to check CRP prior to initiating treatment for ICI in metastatic RCC. The CRP-prognostic tool presented herein is intended for its prognostic value for immunotherapy rather than being predictive to different types of treatments. This study identified strong capacity of CRP in predicting OS and PFS, which outperformed the IMDC risk tool comprised of six factors. Patients with CRP levels of 0.5, 5, 40, and 150 mg/L had predicted median 2-year OS probabilities of 96%, 73%, 42%, and 23%, respectively, and 2-year PFS probabilities of 44%, 26%, 14%, and 9%. Such prognostic power highlights that CRP should be considered as a stratification factor for the design of ICI trials and as a marker to provide realistic expectations to patients initiating ICI treatment. Similar findings were identified in the sunitinib-treated cohort consistent with prior studies (30, 31).

A potential study limitation is that the analysis was only focused on patients treated with atezolizumab plus bevacizumab. Confirming the prognostic association of CRP with survival for other ICIs and combination of immunotherapy/kinase inhibitors is a future direction of research. Furthermore, clinical trial inclusion criteria may limit the generalizability of findings to real-world patient populations. For example, IMmotion150 and IMmotion151 were restricted to patients with Karnofsky performance score ≥70, no history of autoimmune diseases, and no active hepatitis B/C infection or significant cardiovascular disease (14, 15). Future research should validate the prognostic association of CRP in real-world populations to allow implementation of CRP prognostic model for mRCC in the clinic.

## Conclusion

CRP was identified as the most prognostic marker of OS and PFS outcomes for patients with mRCC treated with atezolizumab plus bevacizumab. The study highlights that CRP could be considered as a stratification factor for immunotherapy trials and explored as a prognostic tool for mRCC in the clinic.

## Data availability statement

The data analyzed in this study is subject to the following licenses/restrictions: Data was accessed according to Hoffmann-La Roche policy and has been made available through Vivli, Inc (www.vivli.org). Requests to access these datasets should be directed to Vivli, Inc (www.vivli.org).

## Ethics statement

The studies involving human participants were reviewed and approved by Data was accessed according to Hoffmann-La Roche policy and has been made available through Vivli, Inc (www.vivli.org). Secondary analysis of de-identified data was confirmed exempt from review by the Southern Adelaide Local Health Network, Office for Research and Ethics as it was classified as negligible risk research. The patients/participants provided their written informed consent to participate in this study.

## Authors contributions

AA, JB, AH, MS, RM, GK, and AR contributed to conception, design, assembly of data, data analysis, and interpretation. AA, RM, AH, MS, and AR wrote the main manuscript text. All authors reviewed and approved the manuscript. All authors contributed to the article and approved the submitted version.

## Funding

RM, AR, and MS are supported by the Beat Cancer Research Fellowships from Cancer Council South Australia. AH is supported by a Postdoctoral Fellowship from the National Breast Cancer Foundation, Australia (PF-17-007).

## Acknowledgments

This publication is based on research using de-identified individual participant data from data contributor Hoffmann-La Roche that have been made available through Vivli, Inc. Vivli has not contributed to or approved, and is not in any way responsible for, the contents of this publication.

## Conflict of interest

RM, AR, and MS report investigator-initiated project grants from Pfizer outside the submitted work. AA, JB, GK, and AH have no conflicts of interest to disclose. The funders had no role in the design of the study, in the collection, analyses, or interpretation of data, in the writing of the manuscript or in the decision to publish the results.

The remaining authors declare that the research was conducted in the absence of any commercial or financial relationships that could be construed as a potential conflict of interest.

## Publisher’s note

All claims expressed in this article are solely those of the authors and do not necessarily represent those of their affiliated organizations, or those of the publisher, the editors and the reviewers. Any product that may be evaluated in this article, or claim that may be made by its manufacturer, is not guaranteed or endorsed by the publisher.
