# Supplementary material for: C-reactive protein provides superior prognostic accuracy than the IMDC risk model in renal cell carcinoma treated with Atezolizumab/Bevacizumab
Source: Front Oncol. 2022 Aug 1;12:918993. doi: 10.3389/fonc.2022.918993 (PMC9376479; doi:10.3389/fonc.2022.918993)
Supplement: Supplementary file 1 [file DataSheet_1.docx]

# Supplementary Tables and Figures

Supplementary Table 1. Baseline patients’ characteristics in the atezolizumab plus bevacizumab treated cohorts

| **Variable** | **Total No. 552** |
| --- | --- |
| Actual treatment given: Atezolizumab + Bevacizumab | 552 (100%) |
| Age (years) | 62 (56 - 69) |
| Sex | |
| Male | 389 (70%) |
| Female | 163 (30%) |
| Weight (kg) | |
| Median (IQR) | 78 (65 - 90) |
| Missing | 3 (1%) |
| C Reactive Protein (mg/L) | |
| Median (IQR) | 6.3 (2.1 - 29.4) |
| Missing | 25 (4.5%) |
| Haemoglobin (g/L) | |
| Median (IQR) | 131 (118 - 144) |
| Missing | 5 (1%) |
| Haemoglobin | |
| < LLN | 214 (39%) |
| ≥ LLN | 333 (60%) |
| Missing | 5 (1%) |
| Calcium, Corrected (mmol/L) | |
| Median (IQR) | 2.4 (2.3 - 2.4) |
| Missing | 111 (20.1%) |
| Calcium, Corrected | |
| ≤ULN | 374 (68%) |
| >ULN | 67 (12%) |
| Missing | 111 (20%) |
| Lactate Dehydrogenase (U/L) | |
| Median (IQR) | 186 (156 - 290) |
| Missing | 10 (2%) |
| Lactate Dehydrogenase | |
| ≤ ULN | 445 (81%) |
| > ULN | 97 (18%) |
| Missing | 10 (2%) |
| Neutrophils (10^9/L) | |
| Median (IQR) | 4.6 (3.7 - 5.9) |
| Missing | 9 (1.6%) |
| Neutrophils | |
| ≤ ULN | 477 (86%) |
| > ULN | 66 (12%) |
| Missing | 9 (2%) |
| Platelets (10^9/L) | |
| Median (IQR) | 259 (208 - 326) |
| Missing | 5 (1%) |
| Platelets | |
| ≤ ULN | 479 (87%) |
| > ULN | 68 (12%) |
| Missing | 5 (1%) |
| ECOG PS | |
| 0 | 215 (39%) |
| 1+ | 335 (61%) |
| Missing | 2 (<1%) |
| IMDC risk group | |
| Favourable | 134 (24%) |
| Intermediate | 342 (62%) |
| Poor | 76 (14%) |
| MSKCC (Motzer) risk group | |
| Favourable | 118 (21%) |
| Intermediate | 372 (67%) |
| Poor | 62 (11%) |
| Data are median (IQR) or number of patients (%).P values per Chi-Square test for categorical data and Kruskal-Wallis test for continuous data. LLN: lower limit of normal, ULN: Upper limit of normal | |

Supplementary Table 2. Baseline patients’ characteristics in the atezolizumab plus bevacizumab treated cohorts by C-reactive protein (CRP) groups

| **Variable** | **Total No. 527** | **CRP < 5 mg/L** | **CRP ≥ 5 <30 mg/L** | **CRP ≥ 30 mg/L** | **P-value** |
| --- | --- | --- | --- | --- | --- |
| Actual treatment given: Atezolizumab + Bevacizumab | 527 (100%) | 225 (100%) | 172 (100%) | 130 (100%) |  |
| Age (years) | 62 (56 - 69) | 62 (55 - 69) | 62 (57 - 69) | 62 (56 - 69) | 0.94 |
| Sex | | | | | 0.91 |
| Male | 373 (71%) | 157 (70%) | 123 (72%) | 93 (72%) |  |
| Female | 154 (29%) | 68 (30%) | 49 (28%) | 37 (28%) |  |
| Weight (kg) | | | | | 0.002 |
| Median (IQR) | 78 (66 - 91) | 79 (68 - 91) | 80 (67 - 94) | 75 (63 - 85) |  |
| Missing | 2 (<1%) | 0 (0%) | 0 (0%) | 2 (2%) |  |
| Hemoglobin (g/L) | | | | | < 0.001 |
| Median (IQR) | 131 (118 - 144) | 138 (128 - 148) | 132 (122 - 145) | 112 (102 - 126) |  |
| Missing | 5 (1%) | 1 (<1%) | 3 (2%) | 1 (1%) |  |
| Hemoglobin | | | | | < 0.001 |
| < LLN | 207 (39%) | 43 (19%) | 66 (38%) | 98 (75%) |  |
| ≥ LLN | 315 (60%) | 181 (80%) | 103 (60%) | 31 (24%) |  |
| Missing | 5 (1%) | 1 (<1%) | 3 (2%) | 1 (1%) |  |
| Calcium, Corrected (mmol/L) | | | | | < 0.001 |
| Median (IQR) | 2.4 (2.3 - 2.5) | 2.3 (2.2 - 2.4) | 2.4 (2.3 - 2.4) | 2.4 (2.3 - 2.6) |  |
| Missing | 109 (20.7%) | 48 (21.3%) | 36 (20.9%) | 25 (19.2%) |  |
| Lactate Dehydrogenase (U/L) | | | | | < 0.001 |
| Median (IQR) | 186 (156 - 287) | 174 (152 - 227) | 186 (159 - 286) | 251 (166 - 353) |  |
| Missing | 10 (2%) | 4 (2%) | 3 (2%) | 3 (2%) |  |
| Lactate Dehydrogenase | | | | | < 0.001 |
| ≤ ULN | 424 (80%) | 205 (91%) | 136 (79%) | 83 (64%) |  |
| > ULN | 93 (18%) | 16 (7%) | 33 (19%) | 44 (34%) |  |
| Missing | 10 (2%) | 4 (2%) | 3 (2%) | 3 (2%) |  |
| Neutrophils (10^9/L) | | | | | < 0.001 |
| Median (IQR) | 4.6 (3.7 - 5.9) | 4.1 (3.3 - 5.1) | 4.8 (3.9 - 5.9) | 5.7 (4.4 - 7.2) |  |
| Missing | 8 (1.5%) | 1 (0.4%) | 6 (3.5%) | 1 (0.8%) |  |
| Neutrophils | | | | | < 0.001 |
| ≤ ULN | 456 (87%) | 215 (96%) | 145 (84%) | 96 (74%) |  |
| > ULN | 63 (12%) | 9 (4%) | 21 (12%) | 33 (25%) |  |
| Missing | 8 (2%) | 1 (<1%) | 6 (3%) | 1 (1%) |  |
| Platelets (10^9/L) | | | | | < 0.001 |
| Median (IQR) | 260 (208 - 326) | 234 (188 - 288) | 259 (217 - 320) | 332 (275 - 428) |  |
| Missing | 5 (1%) | 1 (<1%) | 3 (2%) | 1 (1%) |  |
| Platelets | | | | | < 0.001 |
| ≤ ULN | 455 (86%) | 217 (96%) | 144 (84%) | 94 (72%) |  |
| > ULN | 67 (13%) | 7 (3%) | 25 (15%) | 35 (27%) |  |
| Missing | 5 (1%) | 1 (<1%) | 3 (2%) | 1 (1%) |  |
| ECOG PS | | | | | < 0.001 |
| 0 | 206 (39%) | 114 (51%) | 61 (35%) | 31 (24%) |  |
| 1+ | 319 (61%) | 111 (49%) | 110 (64%) | 98 (75%) |  |
| Missing | 2 (<1%) | 0 (0%) | 1 (1%) | 1 (1%) |  |
| IMDC risk group | | | | | < 0.001 |
| Favorable | 127 (24%) | 90 (40%) | 34 (20%) | 3 (2%) |  |
| Intermediate | 327 (62%) | 130 (58%) | 122 (71%) | 75 (58%) |  |
| Poor | 73 (14%) | 5 (2%) | 16 (9%) | 52 (40%) |  |
| MSKCC (Motzer) risk group | | | | | < 0.001 |
| Favorable | 112 (21%) | 76 (34%) | 32 (19%) | 4 (3%) |  |
| Intermediate | 355 (67%) | 147 (65%) | 123 (72%) | 85 (65%) |  |
| Poor | 60 (11%) | 2 (1%) | 17 (10%) | 41 (32%) |  |
| Data are median (IQR) or number of patients (%). P values per Chi-Square test for categorical data and Kruskal-Wallis test for continuous data. LLN: lower limit of normal, ULN: Upper limit of normal, CRP: C-reactive protein | | | | | |

Supplementary Table 3. Prediction performance and effect size of the association of C-reactive protein and IDMC risk tool with overall survival and progression-free survival for patients treated with sunitinib.

|  | **Overall survival** | | | | | **Progression free survival** | | | | |
| --- | --- | --- | --- | --- | --- | --- | --- | --- | --- | --- |
|  | **n** | **HR** | **95% CI** | **P-value** | **c** | **n** | **HR** | **95% CI** | **P-value** | **c** |
| Log C-reactive protein (mg/L) | 509 | 1.78 | 1.60 to 1.99 | <0.001 | 0.76 | 509 | 1.36 | 1.27 to 1.45 | <0.001 | 0.66 |
| IMDC risk group | 546 |  |  | <0.001 | 0.71 | 546 |  |  | <0.001 | 0.62 |
| Favorable |  | 1.00 |  |  |  |  | 1.00 |  |  |  |
| Intermediate |  | 3.30 | 1.81 to 6.04 |  |  |  | 1.72 | 1.29 to 2.29 |  |  |
| Poor |  | 15.1 | 8.09 to 28.1 |  |  |  | 3.91 | 2.80 to 5.47 |  |  |
| Log Hemoglobin (g/L) | 542 | 0.04 | 0.01 to 0.09 | <0.001 | 0.71 | 542 | 0.13 | 0.07 to 0.26 | <0.001 | 0.61 |
| Log Neutrophils (10^9/L) | 537 | 4.17 | 2.73 to 6.37 | <0.001 | 0.65 | 537 | 1.87 | 1.41 to 2.49 | <0.001 | 0.58 |
| ECOG PS | 543 | 2.55 | 1.97 to 3.30 | <0.001 | 0.65 | 543 | 1.31 | 1.10 to 1.56 | 0.003 | 0.55 |
| Log Platelets (10^9/L) | 541 | 5.80 | 3.83 to 8.78 | <0.001 | 0.68 | 541 | 2.52 | 1.88 to 3.36 | <0.001 | 0.59 |
| Log Calcium, Corrected (mmol/L) | 438 | 121 | 13.5 to 1083 | <0.001 | 0.66 | 438 | 1.98 | 0.94 to 4.17 | 0.074 | 0.59 |
| C-reactive protein group (mg/L) |  |  |  | <0.001 | 0.75 |  |  |  | <0.001 | 0.62 |
| < 5 | 225 | 1.00 |  |  |  | 225 | 1.00 |  |  |  |
| ≥ 5 and < 30 | 172 | 3.79 | 2.33 to 6.18 |  |  | 172 | 1.52 | 1.17 to 1.97 |  |  |
| ≥ 30 | 130 | 9.64 | 6.03 to 15.4 |  |  | 130 | 2.79 | 2.14 to 3.65 |  |  |

CI=confidence interval, HR=hazard ratio, OS=overall survival, PFS=progression free survival, IMDC=International Metastatic renal cell carcinoma Database Consortium, n= number of patients, ECOG PS=Eastern Cooperative Oncology Group Performance Status.

Supplementary Table 4. Association of C-reactive protein with objective response rate for patients treated with atezolizumab.

|  | **Events/Patients (%)** | **OR [95% CI]** | **P** |
| --- | --- | --- | --- |
| **C-reactive Protein (mg/L**) |  |  | 0.004 |
| < 5 | 93/225 (41%) | 1.00 |  |
| ≥ 5 < 30 | 66/172 (38%) | 0.88 [0.59-1.33] |  |
| ≥ 30 | 32/130 (25%) | 0.46 [0.29-0.75] |  |

OR: Odds ratio

| 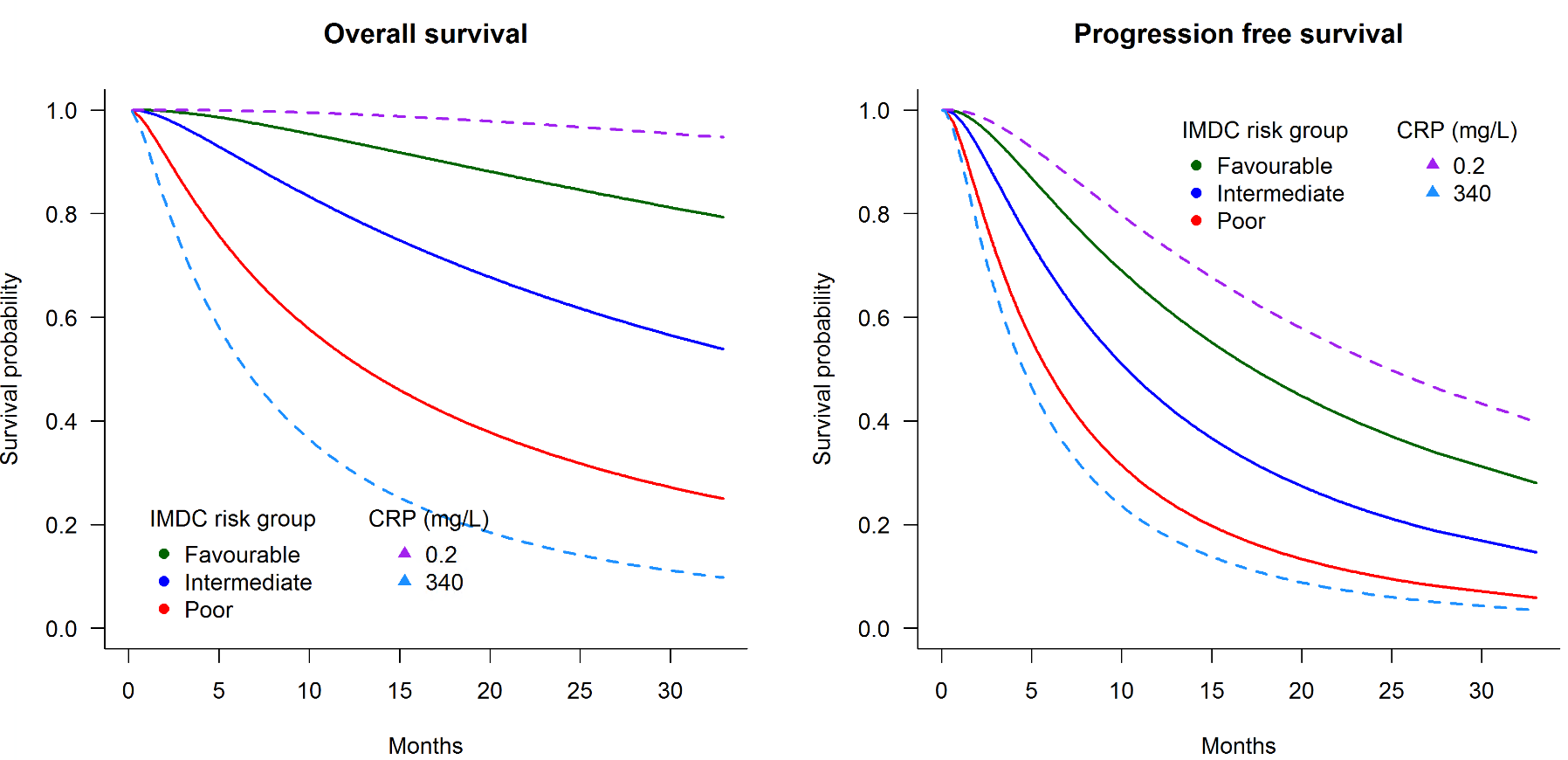 |
| --- |
| Supplementary Figure 1. Predicted survival curves according to IMDC risk groups (solid lines) and pre-treatment C-reactive protein levels (dashed line) for patients treated with atezolizumab. |

| 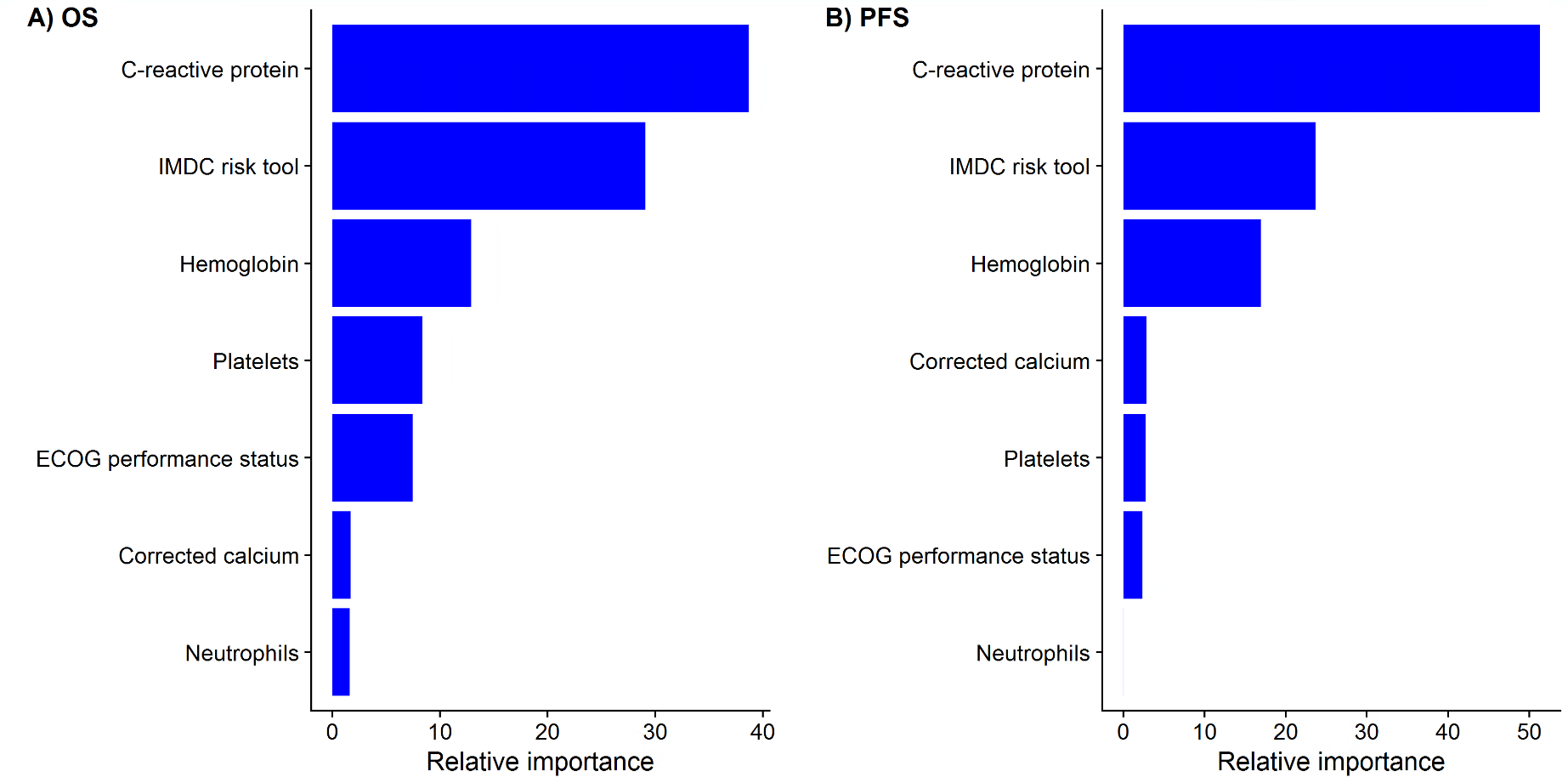 |
| --- |
| Supplementary Figure 2. Relative importance of C-creative protein to IMDC factors for predicting overall survival (OS) and progression-free survival (PFS) using random forest for patients treated with sunitinib. |

| 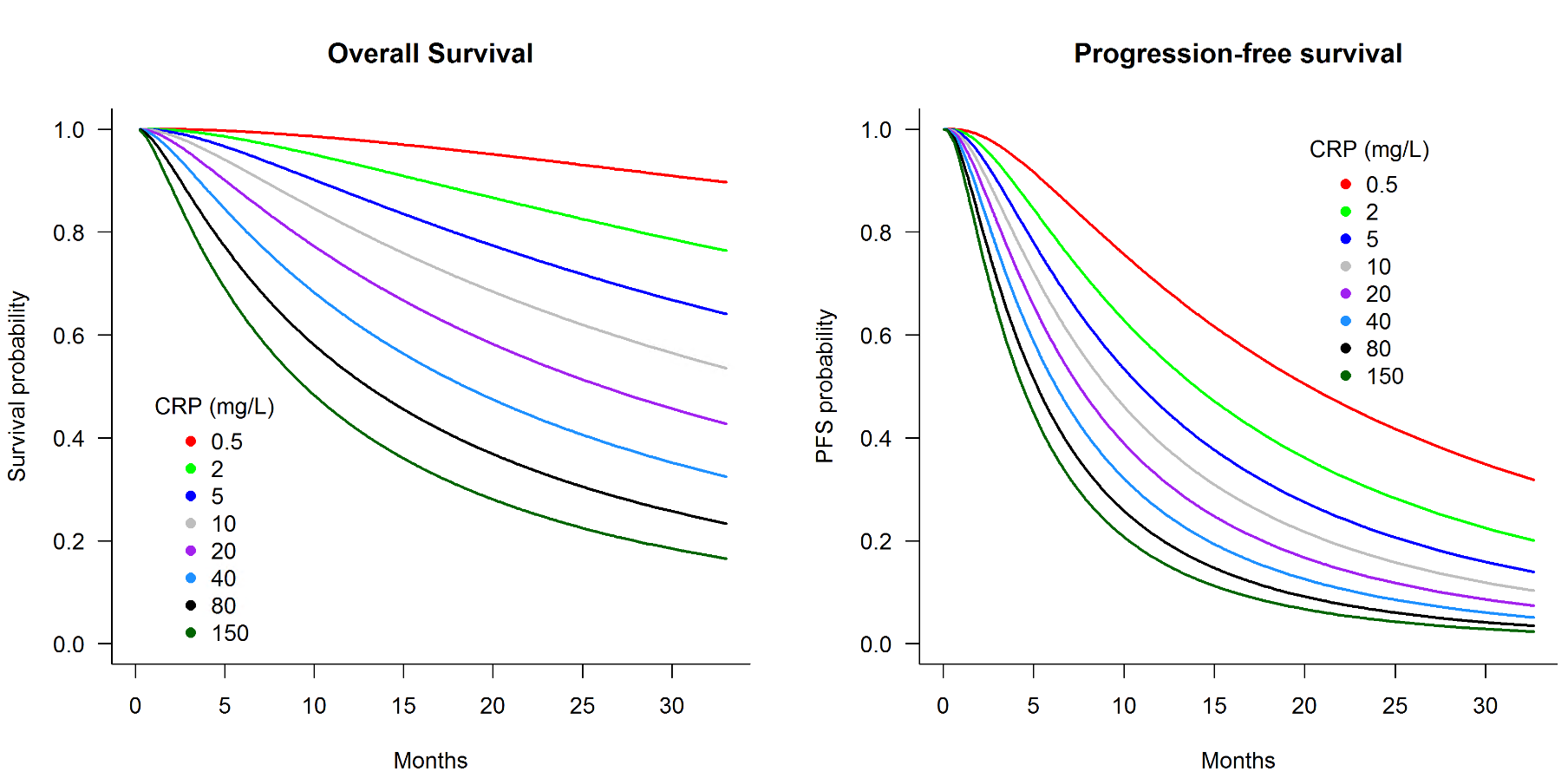 |
| --- |
| Supplementary Figure 3. Predicted survival curves according to pre-treatment C-reactive protein level for patients treated with sunitinib. |

| 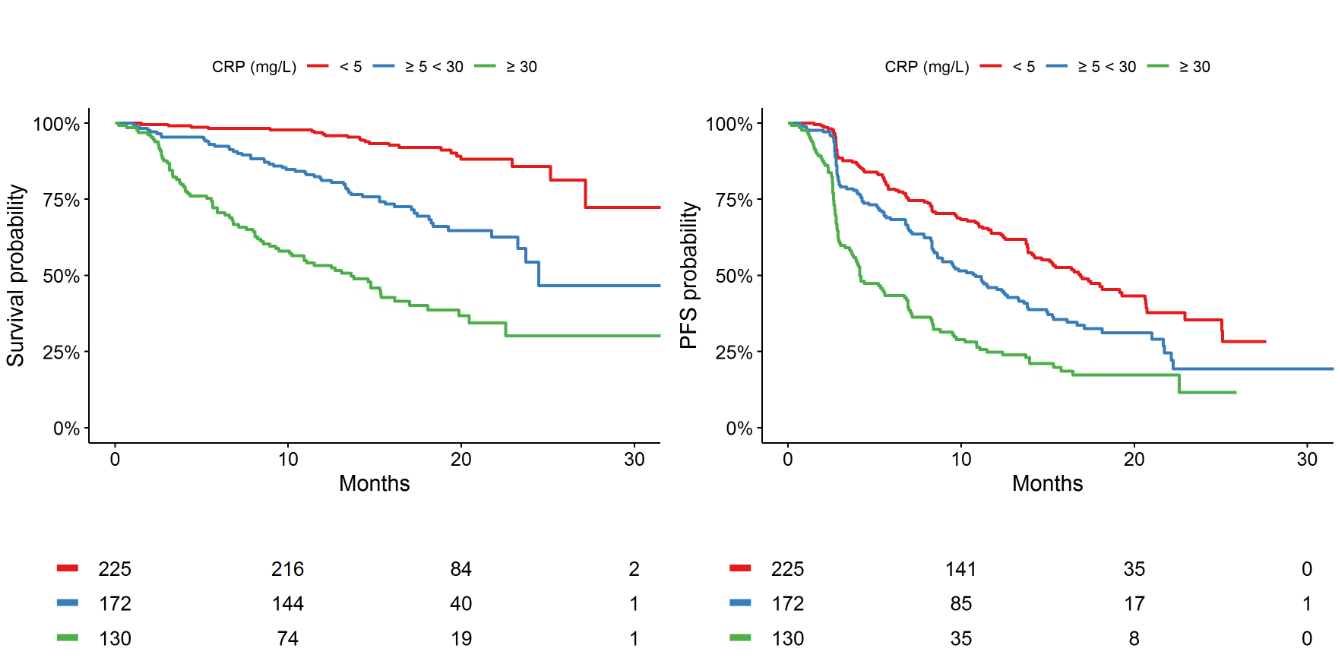 |
| --- |
| Supplementary Figure 4. Kaplan Meier estimates of overall survival by pre-treatment CRP group for patients treated with atezolizumab plus bevacizumab. |
